# Cardiovascular Risk Factors in Childhood and Adolescence

**DOI:** 10.3390/jcm11041136

**Published:** 2022-02-21

**Authors:** Annette Wacker-Gussmann, Renate Oberhoffer-Fritz

**Affiliations:** 1 Institute of Preventive Pediatrics, Faculty of Sport and Health Sciences, Technical University of Munich, 80992 Munich, Germany; renate.oberhoffer@tum.de; 2 German Heart Center, Department of Congenital Heart Disease and Pediatric Cardiology, 80636 Munich, Germany

According to the World Health Organization (WHO), cardiovascular diseases (CVDs) are the leading cause of death globally, taking an estimated amount of 17.9 million lives each year. CVD is therefore a major public health problem contributing to the global burden of morbidity and mortality. It affects people over their whole lifespan, especially the elderly.

Nowadays, cardiovascular (CV) risk factors are dramatically increasing in children and adolescents due to sociodemographic changes. The ongoing COVID-19 pandemic situation even worsens such developments. Physical distancing, distant learning and virtual schooling, exposure to increased screen time, and less physical activity have all contributed to increased weight gain among youth. Weight gain and obesity are associated with increased risk for atherosclerosis and, consequently, CVD in all ages. 

During the Life Course, there is also an accumulation of different risk factors contributing to the development of CVD: in addition to biological and behavioral factors, environmental and psychosocial factors affect well-being and CV health. Moreover, there is the role of the whole family with particular socio-economic and lifestyle factors, which also have to be considered.

In addition to these growing risk factors for acquired CVD in youth, there is still congenital heart disease (CHD) as the most common birth defect worldwide, affecting nearly one percent of newborns each year. With the development of better surgical techniques, infant mortality rates have dropped dramatically. This means that there is a growing population of adults with CHD. Therefore, environmental and lifestyle factors also influence patients with CHD and other chronic diseases, but this is outside of the research focus.

This Special Issue published in the *Journal of Clinical Medicine* aimed to collect high-quality studies that represent the most recent advancements in CV research of children. We focused on different aspects of cardiovascular risks for CVD in childhood and adolescence, from pregnancy and fetal counseling [1] over childhood after high-risk pregnancies [2] to young adults. 

According to the key topics of Pediatric Cardiology, contributions to CHD and cardiomyopathies [3,4] as well as acquired heart disease were edited. Lifestyle factors, nutrition and physical activity [5,6] partly resulting in CV risk factors, such as obesity [7,8], were reported. Bjelakovic et al. [8] published a Position Paper from Serbia, which reported on the CV risk assessment and clinical management of children and adolescents with heterozygous familial hypercholesterolemia, which certainly has a great impact on CVD. 

Multiple interrelated influences with exponentially growing adverse CV effects over the lifespan impede research and raise the question: at which key stage of the life course does the prevention of CV risks in youth achieve the highest benefit for health? It has to be considered that prevention strategies in youth will not only reach adulthood of the individuum itself. Due to the upcoming reproductive age, it will also at least reach the next generation. High-risk pregnancies with the effect of fetal programming, overweight and obesity, arterial hypertension, dyslipidemia and lifestyle factors such as physical activity, sedentary behavior, nutrition and even social problems influence CV health at all ages. This opens an immense field for CV prevention research.

In conclusion, the article collection included in this Special Issue of the *Journal of Clinical Medicine* shows novel, comprehensive and longitudinal datasets that can open new fascinating research avenues. It highlights the role of personalized early prevention and care of CVD beginning in youth.

We thank the authors, the reviewers and the *Journal of Clinical Medicine* for their enthusiastic work on this important topic.
